# Dietary index for gut microbiota and its inverse association with female infertility: evidence from NHANES 2013–2018

**DOI:** 10.3389/fnut.2025.1564549

**Published:** 2025-04-04

**Authors:** Chiqiang Cheng, Xi He, Chunhua Zhou, Liu Ouyang, Yang Zhao, Jiahui Li, Fangfang Liu, Xia Gao

**Affiliations:** Department of Obstetrics, Renmin Hospital, Hubei University of Medicine, Shiyan, China

**Keywords:** DI-GM, infertility, dietary index for gut microbiota, NHANES, dietary

## Abstract

**Background:**

Infertility has become a global health concern, especially as the aging population continues to grow. Previous studies suggest that gut microbiota plays a crucial role in female reproductive health. This study aimed to investigate the association between the Dietary Index for Gut Microbiota (DI-GM) and female infertility.

**Methods:**

We analyzed data from 8,910 participants aged 20–45 years in the NHANES 2013–2018 cycles. DI-GM scores were calculated based on dietary recall interviews, including 14 foods and nutrients associated with gut health. Female infertility was identified through reproductive health questionnaires. Weighted logistic regression models were used to assess the relationship between DI-GM and infertility, with adjustments for demographic, lifestyle, and health-related covariates. Restricted cubic spline (RCS) analyses explored non-linear associations, and subgroup analyses ensured the robustness of the results.

**Results:**

A significant negative association was observed between DI-GM and female infertility (adjusted OR = 0.89, 95% CI: 0.83–0.95, *p* < 0.001). Participants with DI-GM scores ≥6 had a 40% lower risk of infertility compared to those with scores 0–3 (OR = 0.60, 95% CI: 0.44–0.82, *p* = 0.001). RCS analyses revealed an L-shaped non-linear relationship, with a threshold at DI-GM = 5. Subgroup analyses indicated stronger associations in women with lower education levels and those with coronary heart disease (*P* for interaction <0.05).

**Conclusion:**

Our findings demonstrate that a higher DI-GM score is associated with a reduced risk of female infertility, suggesting that dietary interventions targeting gut microbiota may offer a cost-effective strategy for improving reproductive health. Further longitudinal and interventional studies are warranted to confirm causality and elucidate underlying mechanisms.

## 1 Background

Population aging has become a global trend. According to a United Nations report, by 2050, the number of people aged 60 years and older will reach 2 billion, accounting for 22% of the global population (1). As the aging population increases, fertility-related issues have become more pronounced, drawing significant attention from scholars worldwide. Female infertility can result from various factors, including tubal disorders, uterine abnormalities, endocrine dysfunction, and immune system imbalances (2, 3). Additionally, women’s fertility declines significantly after the age of 35. Although assisted reproductive technologies (ART), such as *in vitro* fertilization (IVF) and intrauterine insemination (IUI), can address ovulatory dysfunction and tubal obstruction, these treatments impose considerable financial and healthcare burdens (3, 4). Luke et al. (4) reported that the cost of ART in the United States ranges from $12,000 to $15,000 per cycle, with older patients typically requiring 2–3 cycles to achieve pregnancy. As women age, ovarian reserves diminish, and embryo quality declines. Evers (5) also noted that the success rate of IVF in women aged 35–37 is approximately 35%, whereas it drops to around 20% for women over 40. Consequently, older women face psychological stress from repeated pregnancy failures. Beyond psychological challenges, infertility increases the risk of several diseases, including cardiovascular diseases, diabetes, and metabolic syndrome (6). Addressing female infertility effectively can enhance fertility rates and significantly reduce the overall disease and economic burden associated with these conditions.

Recent studies have highlighted the pivotal role of gut microbiota in female reproductive health. Dysbiosis of the gut microbiota has been linked to several infertility-related conditions, including polycystic ovary syndrome (PCOS), endometriosis, and obesity (7, 8). Dietary modulation of the gut microbiota can indirectly improve fertility by optimizing estrogen metabolism, reducing inflammation, and enhancing metabolic health (9). Studies have shown that adherence to the Mediterranean diet increases the likelihood of successful pregnancies in infertile women by 28% (10). Zhang et al. (11) found that foods rich in vitamin D and omega-3 fatty acids reduce the incidence of PCOS and improve ovulation rates (12). Research by Kase et al. (13) demonstrated that *Bifidobacterium* and *Lactobacillus* optimize estrogen metabolism by reducing toxic gut metabolites, lowering *β*-glucuronidase activity, and strengthening intestinal epithelial tight junctions. Additionally, *Akkermansia muciniphila*, *Bifidobacterium bifidum*, and *Lactiplantibacillus plantarum* improve gut-liver axis function, promote short-chain fatty acid (SCFA) production, and modulate inflammatory responses, thereby influencing estrogen metabolism (14). Thus, a balanced gut microbiota not only profoundly impacts female reproductive health but also offers a novel direction for infertility treatment.

Optimizing dietary patterns is now recognized as a key strategy for improving gut microbiota composition. Numerous studies have confirmed that the consumption of specific foods—such as fermented dairy products, chickpeas, soy, whole grains, cranberries, and green tea—enhances gut microbial diversity, increases SCFA synthesis, and improves the Firmicutes-to-Bacteroidetes ratio (15–17). However, there remains a lack of quantitative tools to assess gut microbiota diversity based on individual dietary patterns. To address this gap, Kase et al. (13) developed and validated the Dietary Index for Gut Microbiota (DI-GM). This index incorporates 14 foods and nutrients, with beneficial components including fermented dairy products, chickpeas, soy, whole grains, dietary fiber, cranberries, avocados, broccoli, coffee, and green tea. Conversely, unfavorable components include refined grains, red meat, processed meat, and high-fat diets (≥40% energy from fat). Dietary interventions offer a cost-effective and feasible strategy for health improvement, making the study of DI-GM and its association with female infertility particularly relevant.

The DI-GM score has been shown to reflect the relationship between dietary patterns and gut microbiota diversity. However, its application in infertility research remains limited. This study aims to explore the potential association between DI-GM and female infertility using data from the NHANES 2013–2018 cycles.

## 2 Materials and methods

### 2.1 Data sources and study population

**Figure 1:**
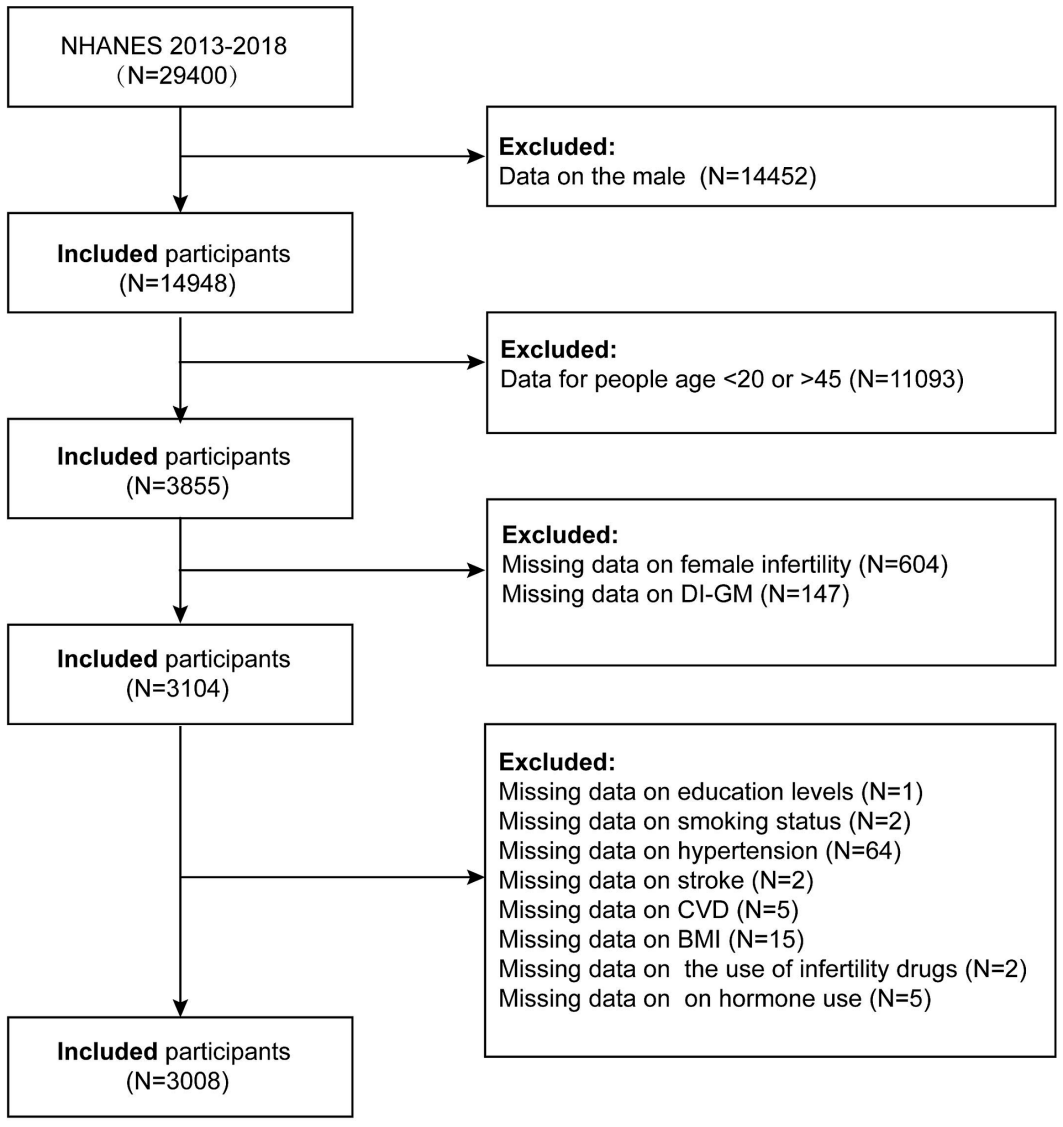
Flow chart of the sample selection from NHANES 2013–2018.

This study utilized publicly available data from the National Health and Nutrition Examination Survey (NHANES), conducted by the National Center for Health Statistics (NCHS) under the Centers for Disease Control and Prevention (CDC). We analyzed data from the 2013–2018 NHANES cycles, initially including 30,008 participants. The exclusion criteria were as follows: (1) male participants (*n* = 14,452); (2) participants under 20 years or over 45 years of age (*n* = 11,093); (3) those with missing data on female infertility (*n* = 604); (4) missing DI-GM data (*n* = 147); (5) missing educational level data (*n* = 1); (6) missing smoking status (*n* = 2); (7) missing hypertension data (*n* = 64); (8) missing stroke data (*n* = 2); (9) missing cardiovascular disease data (*n* = 5); (10) missing body mass index (BMI) data (*n* = 15, 11) missing infertility medication data (*n* = 2); and (12) missing female hormone data (*n* = 5). Ultimately, 3,008 participants were included in the final analysis (Figure 1).

### 2.2 Definition of DI-GM

In NHANES, two dietary recall interviews were conducted. The first evaluation was a 24-h dietary recall interview at the MEC, and the second evaluation was a telephone interview, which recorded the comprehensive and detailed information on participants’ dietary intake in the past 24 h, including all foods and drinks. According to research by Kase et al. (13), 14 kinds of foods and nutrients were finally included in the DI-GM score, comprising 10 foods that promote gut health and 4 that are detrimental to gut health (18). For beneficial foods, participants with an intake exceeding the sex-specific median are assigned a value of 1, while those below the median receive a value of 0. In contrast, for unfavorable foods, an intake above the sex-specific median is assigned a value of 0 and a value of 1 is given to those below the median. The DI-GM score is determined by adding the scores from each section, with a range between 0 and 14. A higher DI-GM score indicates a healthier gut microbiota (19).

### 2.3 Definition of infertility

Infertility was defined based on the reproductive health questionnaire from the NHANES database (files RHQ074 and RHQ076). Participants were asked, “Have you ever tried to get pregnant for at least a year without success?” or “Have you ever seen a doctor or other health care provider because you were unable to get pregnant?” Those who answered “yes” to either question were classified as having female infertility. Previous studies have validated the robustness of this definition (20, 21).

### 2.4 Covariates

In this study, covariates included age and race/ethnicity (Mexican American, other Hispanic, non-Hispanic White, non-Hispanic Black, and other races). Educational attainment was categorized as less than high school, high school, and more than high school. Marital status was classified into never married, married/living with a partner, and widowed/divorced/separated. The poverty income ratio (PIR) was divided into three groups: <1.3 (low income), 1.3–3.5 (middle income), and > 3.5 (high income). Body mass index (BMI) was categorized into <25 kg/m^2^, 25–30 kg/m^2^, and ≥ 30 kg/m^2^. Smoking status was defined as current smokers (≥100 cigarettes in a lifetime and currently smoking), former smokers (≥100 cigarettes in a lifetime but no longer smoking), and never smokers (<100 cigarettes in a lifetime or never smoked). Alcohol consumption was defined as drinking more than 12 times in any year of life. Diabetes was defined as a physician diagnosis or fasting blood glucose level ≥ 126 mg/dL. Hypertension was determined by a previous diagnosis, current antihypertensive medication use, or an average of three blood pressure readings ≥ 140/90 mmHg. Stroke was categorized as yes or no. Cardiovascular disease (CVD) is defined as a history of congestive heart failure, coronary artery disease, angina, or myocardial infarction. Additional covariates included whether participants had regular menstrual periods in the past 12 months, received treatment for pelvic inflammatory disease (PID), underwent hysterectomy, had bilateral oophorectomy, took birth control pills, or used female hormones.

### 2.5 Statistical analyses

The statistical analysis followed the NHANES data analysis guidelines, incorporating appropriate NHANES complex multistage sampling weights. For continuous variables, survey-weighted means with 95% confidence intervals (CIs) were reported, while survey-weighted percentages with 95% CIs were provided for categorical variables. Differences between the infertility and non-infertility groups were assessed using weighted linear regression or weighted chi-square tests.

Three weighted logistic regression models were employed to examine the association between the Dietary Index for Gut Microbiota (DI-GM) and female infertility. Model 1 was unadjusted. Model 2 adjusted for age, race/ethnicity, education level, and marital status. Model 3 further adjusted for additional covariates, including BMI, PIR, smoking status, alcohol consumption, diabetes, hypertension, CVD, stroke, regular menstrual cycles, pelvic inflammatory disease, hysterectomy, bilateral oophorectomy, use of birth control pills, and use of female hormones.

Restricted cubic spline (RCS) analysis was conducted to investigate potential nonlinear associations between DI-GM and female infertility after adjusting for covariates. Additionally, subgroup analyses and interaction tests were performed to explore variations in the association across different subgroups based on potential confounders listed in the baseline table. Statistical analyses were conducted using R software (version 4.3.3), with statistical significance set at *p* < 0.05.

## 3 Results

### 3.1 Baseline characteristics of the study population

A total of 3,008 participants were included in this study. The weighted prevalence of female infertility was 14.10% (95% CI: 12.56–15.78%), with a weighted mean age of 35.37 years (95% CI: 34.44–36.31). Compared to women without infertility, those with infertility had significantly lower DI-GM scores (4.72, 95% CI: 4.48–4.96 vs. 5.04, 95% CI: 4.94–5.15, *p* < 0.01).

In addition, women in the infertility group were more likely to be Non-Hispanic White, widowed/divorced/separated, have a BMI ≥ 30 kg/m^2^, smoke, and have a history of hypertension, diabetes, coronary heart disease, pelvic infections, use of birth control pills, and hormone therapy. Significant differences between the infertility and non-infertility groups were observed across these variables (*p* < 0.01) (Table 1).

**Table 1:** Weighted baseline characteristics of the study population, 2013–2018.

Characteristics	Total (*n* = 3008)	Non-infertility (*n* = 2619)	Infertility (*n* = 389)	*p*-value
Age (years)	32.28 (31.87, 32.69)	31.77 (31.36, 32.18)	35.37 (34.44, 36.31)	<0.0001
Race (%)				0.0041
Mexican American	11.66 (9.02, 14.95)	11.98 (9.28, 15.33)	9.77 (6.26, 14.94)	
Other Hispanic	7.79 (6.29, 9.61)	8.26 (6.74, 10.07)	4.94 (2.98, 8.08)	
Non-Hispanic White	56.89 (51.96, 61.68)	55.54 (50.52, 60.45)	65.07 (57.98, 71.54)	
Non-Hispanic Black	13.37 (10.91, 16.30)	13.64 (11.04, 16.73)	11.75 (8.92, 15.33)	
Other race	10.29 (8.72, 12.10)	10.58 (8.92, 12.52)	8.48 (6.29, 11.33)	
Education level (%)				0.6448
Less than high school	11.28 (9.65, 13.15)	11.54 (9.84, 13.48)	9.71 (6.95, 13.39)	
High school	19.11 (16.80, 21.65)	19.07 (16.86, 21.51)	19.33 (14.04, 26.00)	
More than high school	69.61 (66.12, 72.89)	69.39 (65.83, 72.74)	70.97 (64.40, 76.76)	
Marital status (%)				<0.0001
Never married	29.67 (27.13, 32.34)	32.53 (29.81, 35.38)	12.22 (9.35, 15.83)	
Married/Living with partner	60.24 (57.46, 62.95)	57.57 (54.75, 60.35)	76.48 (71.19, 81.06)	
Widowed/divorced/Separated	10.09 (8.73, 11.64)	9.90 (8.33, 11.72)	11.29 (7.65, 16.36)	
PIR (%)				0.1059
<1.3	29.50 (26.67, 32.49)	30.29 (27.36, 33.38)	24.69 (19.80, 30.33)	
1.3–3.5	36.37 (33.79, 39.03)	36.24 (33.66, 38.90)	37.16 (31.39, 43.32)	
≥3.5	34.13 (30.69, 37.76)	33.47 (29.98, 37.17)	38.16 (31.53, 45.26)	
BMI (%)				0.0011
<25	35.40 (32.83, 38.06)	36.48 (33.69, 39.37)	28.83 (23.13, 35.28)	
25–30	24.13 (22.46, 25.88)	24.95 (23.10, 26.90)	19.11 (14.51, 24.75)	
≥30	40.47 (37.98, 43.02)	38.57 (35.94, 41.27)	52.06 (44.15, 59.87)	
Smoking status (%)				0.0428
Never	67.84 (65.31, 70.28)	68.95 (66.38, 71.39)	61.13 (55.69, 66.30)	
Now	19.44 (17.49, 21.55)	18.71 (16.89, 20.67)	23.91 (18.13, 30.84)	
Former	12.72 (11.16, 14.46)	12.35 (10.79, 14.09)	14.96 (10.77, 20.42)	
Alcohol intake (%)				0.9656
No	16.26 (14.22, 18.53)	16.28 (14.18, 18.61)	16.17 (11.85, 21.68)	
Yes	83.74 (81.47, 85.78)	83.72 (81.39, 85.82)	83.83 (78.32, 88.15)	
Hypertension (%)				<0.0001
No	84.55 (82.76, 86.18)	85.95 (84.20, 87.53)	76.04 (70.37, 80.91)	
Yes	15.45 (13.82, 17.24)	14.05 (12.47, 15.80)	23.96 (19.09, 29.63)	
Diabetes (%)				0.0074
No	94.87 (93.93, 95.67)	95.36 (94.28, 96.24)	91.87 (88.78, 94.17)	
Yes	5.13 (4.33, 6.07)	4.64 (3.76, 5.72)	8.13 (5.83, 11.22)	
Stroke (%)				0.4679
No	99.47 (99.07, 99.70)	99.44 (98.97, 99.69)	99.68 (98.62, 99.93)	
Yes	0.53 (0.30, 0.93)	0.56 (0.31, 1.03)	0.32 (0.07, 1.38)	
CVD (%)				0.2599
No	98.67 (98.05, 99.09)	98.79 (98.17, 99.20)	97.93 (95.05, 99.15)	
Yes	1.33 (0.91, 1.95)	1.21 (0.80, 1.83)	2.07 (0.85, 4.95)	
Had regular periods in past 12 months (%)				0.0520
No	11.49 (10.25, 12.85)	10.89 (9.57, 12.36)	15.13 (11.15, 20.21)	
Yes	88.51 (87.15, 89.75)	89.11 (87.64, 90.43)	84.87 (79.79, 88.85)	
Ever treated for a pelvic infection/PID (%)				<0.0001
No	95.29 (94.17, 96.20)	96.14 (95.14, 96.94)	90.13 (85.68, 93.30)	
Yes	4.71 (3.80, 5.83)	3.86 (3.06, 4.86)	9.87 (6.70, 14.32)	
Had a hysterectomy (%)				0.2907
No	95.20 (94.22, 96.03)	95.46 (94.37, 96.34)	93.63 (88.92, 96.42)	
Yes	4.80 (3.97, 5.78)	4.54 (3.66, 5.63)	6.37 (3.58, 11.08)	
Had both ovaries removed (%)				0.1394
No	98.15 (97.33, 98.72)	98.38 (97.45, 98.97)	96.76 (93.08, 98.51)	
Yes	1.85 (1.28, 2.67)	1.62 (1.03, 2.55)	3.24 (1.49, 6.92)	
Ever taken birth control pills (%)				0.0073
No	25.82 (23.48, 28.30)	26.90 (24.51, 29.43)	19.22 (14.50, 25.02)	
Yes	74.18 (71.70, 76.52)	73.10 (70.57, 75.49)	80.78 (74.98, 85.50)	
Ever use female hormones (%)				0.0020
No	95.12 (93.50, 96.35)	96.02 (94.57, 97.10)	89.60 (82.08, 94.19)	
Yes	4.88 (3.65, 6.50)	3.98 (2.90, 5.43)	10.40 (5.81, 17.92)	
DI-GM	5.00 (4.90, 5.10)	5.04 (4.94, 5.15)	4.72 (4.48, 4.96)	0.0075
DI-GM group (%)				0.0311
0–3	18.85 (16.82, 21.06)	17.95 (16.02, 20.06)	24.35 (18.59, 31.20)	
4	20.39 (18.94, 21.91)	19.91 (18.39, 21.52)	23.31 (18.79, 28.53)	
5	24.86 (22.97, 26.85)	25.51 (23.51, 27.62)	20.91 (15.42, 27.71)	
≥6	35.90 (33.28, 38.61)	36.63 (34.01, 39.34)	31.43 (25.49, 38.06)	

PIR, poverty income ratio; BMI, body mass index; CVD, cardiovascular disease; DI-GM, dietary index for gut microbiota; PID, pelvic inflammatory disease.

### 3.2 Association between DI-GM and female infertility

Multivariate logistic regression analysis revealed a negative association between DI-GM and female infertility. After adjusting for all covariates, DI-GM was significantly inversely correlated with infertility (OR = 0.89, 95% CI: 0.83–0.95). Furthermore, when DI-GM was categorized into quartiles, participants with a DI-GM score > 6 had a significantly lower likelihood of infertility compared to those in the lowest quartile (OR = 0.60, 95% CI: 0.44–0.82) (Table 2).

**Table 2:** Multivariate logistic regression between DI-GM and female infertility.

Characteristic	Model 1		Model 2		Model 3	
	OR (95% CI)	*p*-value	OR (95% CI)	*p*-value	OR (95% CI)	*p*-value
DI-GM (continuous)	0.90 (0.85, 0.96)	0.002	0.88 (0.82, 0.94)	<0.001	0.89 (0.83, 0.95)	<0.001
DI-GM (group)						
0–3	Reference		Reference		Reference	
4	0.83 (0.61, 1.13)	0.240	0.79 (0.58, 1.08)	0.145	0.83 (0.60, 1.15)	0.264
5	0.61 (0.44, 0.83)	0.002	0.55 (0.40, 0.76)	<0.001	0.55 (0.40, 0.77)	<0.001
≥6	0.64 (0.48, 0.86)	0.003	0.57 (0.42, 0.77)	<0.001	0.60 (0.44, 0.82)	0.001
*P* for trend		<0.001		<0.001		<0.001

Model 1: No covariates were adjusted. Model 2: Adjusted for age, and race, education level, marital status. Model 3: Adjusted for age, race, education level, marital status, PIR, BMI, smoking status, alcohol consumption, diabetes, hypertension, CVD, stroke, pelvic infection/PID, regular menstrual periods, history of hysterectomy, history of bilateral oophorectomy, female hormones taken, and birth control pills taken.

**Figure 2:**
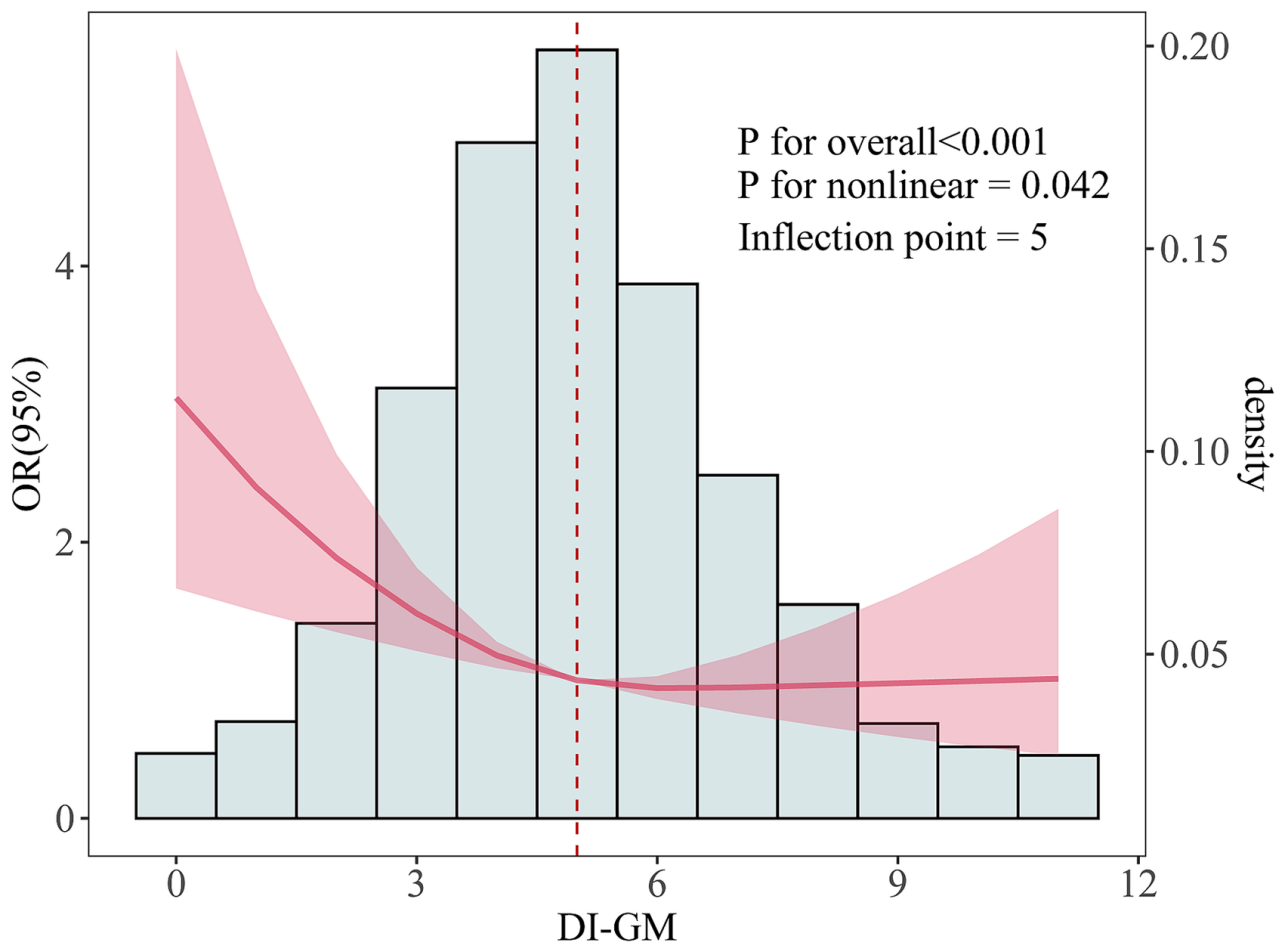
The dose-effect relationship between DI-GM and female infertility. Age, race, education level, marital status, PIR, BMI, smoking status, alcohol consumption, diabetes, hypertension, CVD, stroke, pelvic infection/PID, regular menstrual periods, history of hysterectomy, history of bilateral oophorectomy, female hormones taken, and birth control pills taken were adjusted.

Interestingly, the restricted cubic spline (RCS) analysis demonstrated an L-shaped nonlinear negative association between DI-GM and infertility, with a threshold point at DI-GM = 5 (Figure 2).

### 3.3 Subgroup analyses

**Figure 3:**
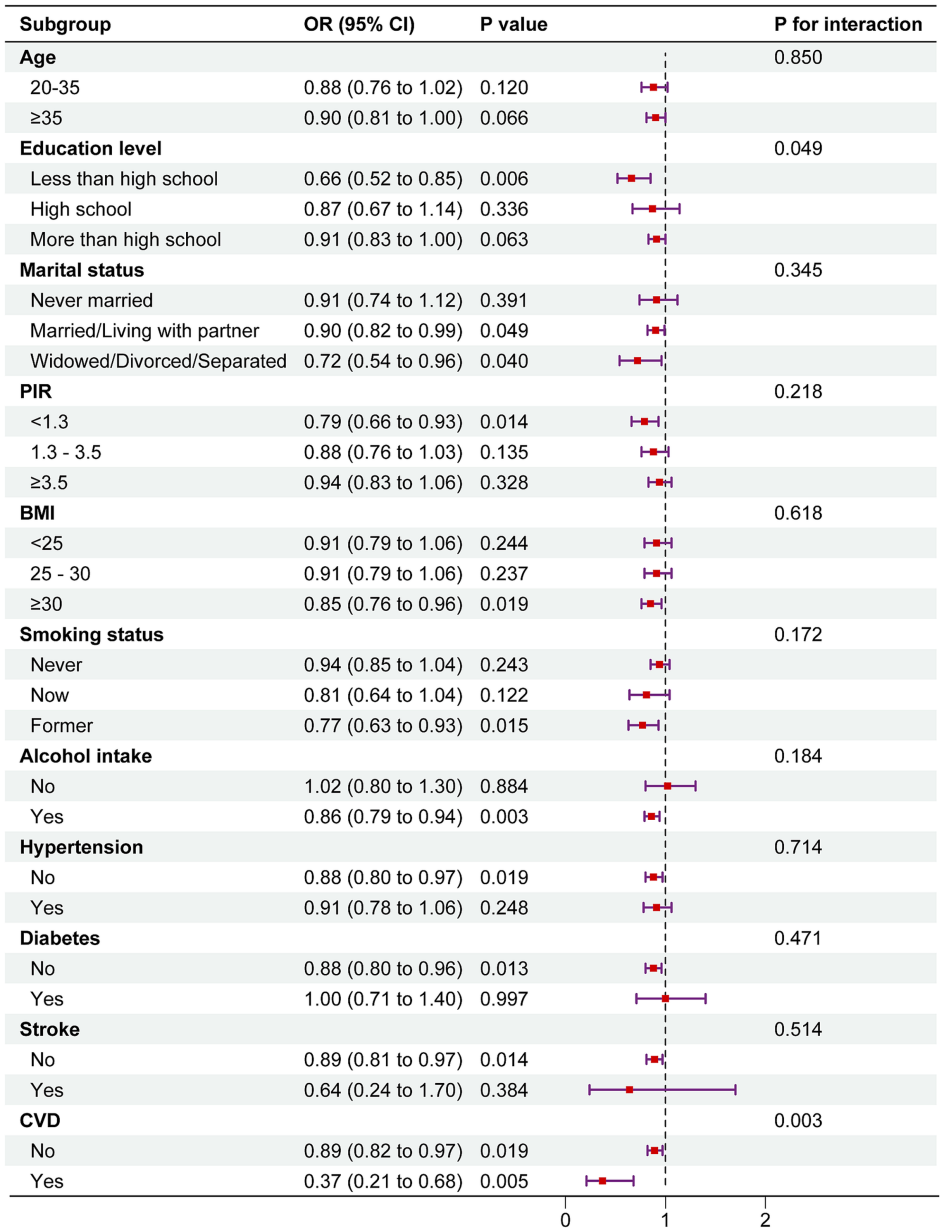
Subgroup analysis of the association between DI-GM and female infertility. The above model was adjusted for age, race, education level, marital status, PIR, BMI, smoking status, alcohol consumption, diabetes, hypertension, CVD, stroke, pelvic infection/PID, regular menstrual periods, history of hysterectomy, history of bilateral oophorectomy, female hormones taken, and birth control pills taken. In each case, the model was not adjusted for the stratification variable.

The results indicated that DI-GM maintained an inverse association with female infertility across all subgroups. Interestingly, significant differences were observed in the association between DI-GM and infertility prevalence within the education level and coronary heart disease (CHD) subgroups (*P* for interaction < 0.05). The association was notably stronger among individuals with less than a high school education (OR = 0.66, 95% CI: 0.52–0.85) and those with CHD (OR = 0.37, 95% CI: 0.21–0.68) (Figure 3).

## 4 Discussion

This study demonstrates that optimizing dietary patterns to improve the Dietary Index for Gut Microbiota (DI-GM) may serve as a significant strategy for reducing the risk of female infertility. Logistic regression analysis revealed a significant inverse association between DI-GM and female infertility. In Model 1, which did not adjust for covariates, each unit increase in DI-GM was associated with a significant reduction in infertility risk (OR = 0.90, 95% CI: 0.85–0.96, *p* = 0.002). This inverse association remained robust even after comprehensive adjustment for confounders, including BMI, smoking status, alcohol consumption, chronic conditions, and use of female hormones, in Model 3 (OR = 0.89, 95% CI: 0.83–0.95, *p* < 0.001). Subgroup analysis further revealed that women with higher DI-GM scores (≥6) exhibited a 40% lower risk of infertility compared to those in the lowest quartile (0–3 points) (OR = 0.60, 95% CI: 0.44–0.82, *p* = 0.001). Additionally, restricted cubic spline analysis identified an L-shaped nonlinear relationship between DI-GM and infertility, with a turning point at DI-GM = 5. Beyond this threshold, the declining trend in infertility risk plateaued. Subgroup and interaction analyses further validated the robustness of these findings.

With global population aging and the trend of delayed childbearing, fertility challenges have become increasingly prominent (22). Female infertility, characterized by its high prevalence and profound impact on both individuals and society, has garnered significant attention (23). According to WHO estimates, approximately 10–15% of women of reproductive age experience infertility (24). A prospective cohort study indicated that delaying the age of first childbirth significantly increases the risk of obesity-related reproductive cancers (25). Similarly, the incidence of cardiovascular events in women with polycystic ovary syndrome (PCOS) is three times higher than in the general population (11). Mental health issues also deserve attention, as studies have reported that the prevalence of depression and anxiety among infertile women reaches 30 and 25%, respectively (26).

In recent years, dietary interventions for infertility have garnered increased attention because they are cost-effective and easy to implement (27). An increasing body of evidence suggests that the gut microbiota not only regulates systemic inflammation and metabolic homeostasis but also plays a crucial role in sex hormone metabolism. Results from our study indicate that a higher DI-GM score (representing a microbiota-friendly dietary pattern) is negatively associated with female infertility, aligning with emerging studies on the gut-hormone axis and its impact on reproductive outcomes (28, 29). In a study involving individuals with polycystic ovary syndrome (PCOS), Wang et al. (45) found that combined probiotic and dietary fiber supplementation significantly enhanced insulin sensitivity and promoted ovulation recovery, thus providing direct evidence of the efficacy of probiotic interventions (30, 31). Similarly, a preclinical study reported elevated abundances of specific bacterial taxa, such as UBA1819 lineage (family *Lachnospiraceae*), *Eisenbergiella*, *Hungatella*, and *Erysipelatoclostridium*, in fecal samples from individuals with endometriosis (32). Notably, supplementation with short-chain fatty acids (e.g., butyrate), produced by microbial fermentation of dietary fiber, substantially improved the endometrial microenvironment and increased embryo implantation rates (33, 34). Another study highlighted that synbiotic administration not only restored gut microbial balance but also significantly reduced depression and anxiety scores in patients with infertility, thereby conferring psychological benefits as well. A key mechanism linking diet, gut microbiota, and fertility is progesterone metabolism, which is essential for endometrial receptivity and embryo implantation. Chen et al. (35) provided experimental evidence that gut bacteria grown in nutrient-rich media (reflecting a high-fat, high-protein diet) exhibited markedly increased progesterone metabolism relative to those cultured in minimally defined media (mimicking a plant-based, low-fat diet). These findings suggest that dietary components can directly modulate microbial activity, thereby influencing circulating progesterone levels. Further *in vivo* research supports this mechanism: transplantation of *nonpathogenic Clostridium* into mouse models enhanced progesterone metabolism and subsequently reduced serum progesterone concentrations (36, 37). Appropriate dietary interventions may thus rapidly remodel gut microbial ecology while holistically improving both reproductive capacity and overall health status in women with infertility.

DI-GM, as a scientifically validated tool, assesses how dietary patterns influence overall health through gut microbiota. It includes 14 foods that play critical roles in modulating the ratio of *Firmicutes* to *Bacteroidetes*, inflammation levels, and gut barrier function (19). These foods commonly enhance gut microbiota diversity and promote the production of short-chain fatty acids (SCFAs) (38). Our study provides preliminary evidence that higher DI-GM scores are inversely associated with female infertility. Specifically, fermented dairy products are rich in *Lactobacillus*, chickpeas, and soy are high in *Bifidobacterium*, whole grains contain *Prevotella*, while cranberries and broccoli are abundant in *Bacteroides* (38, 39). While green tea itself does not contain *Lactobacillus* or *Bifidobacterium*, its bioactive polyphenols, particularly catechins, have been shown to exhibit prebiotic properties, selectively promoting the growth of these beneficial bacteria in the gut. The potential mechanisms underlying these associations include: *Lactobacillus* reducing pH levels in the gut and reproductive tract, lowering chronic inflammation, and minimizing intestinal permeability (40); *Bifidobacterium* optimizing the gut-liver axis and regulating menstrual cycles (41); *Prevotella* enhancing gut barrier function and lipid metabolism to reduce visceral fat accumulation and inflammation (42); and *Bacteroides* mitigating oxidative stress associated with endometriosis, thereby improving the embryo implantation environment (43).

Our subgroup analysis revealed that the association between DI-GM and female infertility was notably stronger among women with lower education levels and those with coronary heart disease (CHD). This finding aligns with prior studies suggesting that socioeconomic factors, particularly education level, play a crucial role in shaping health behaviors and disease risk. Individuals with lower educational attainment may have limited health literacy, making it more challenging to access, interpret, and apply health-related information to their daily lives. As a result, they are less likely to engage in preventive health behaviors, including adherence to healthy dietary patterns, regular physical activity, and routine medical check-ups, all of which are crucial for maintaining cardiovascular and reproductive health. Chronic systemic inflammation and metabolic dysfunction—both prevalent in CHD—have been implicated in endothelial dysfunction, insulin resistance, and hormonal imbalances, which may further contribute to infertility. Emerging evidence also suggests that gut microbiota composition is significantly altered in individuals with both CHD and metabolic syndrome, characterized by a reduction in beneficial bacterial taxa and an increase in pro-inflammatory microbial metabolites, potentially exacerbating cardiovascular and reproductive health decline.

In conclusion, our study systematically evaluated the impact of DI-GM on female infertility and found a significant inverse relationship between higher DI-GM scores and infertility risk. Additionally, restricted cubic spline analysis confirmed an L-shaped nonlinear relationship between DI-GM and infertility risk. Subgroup and interaction analyses revealed stronger associations among women with lower education levels and those with CHD, likely due to higher levels of chronic inflammation and metabolic dysregulation in these groups. These findings suggest that dietary interventions may improve gut microbiota diversity more effectively in certain populations, thereby modulating inflammatory responses and estrogen metabolism more efficiently. However, no significant interactions were observed across different racial and income groups, indicating that metabolic or inflammatory status may play a crucial role in driving these differences.

## 5 Strengths and limitations

A notable strength of this study lies in the utilization of a novel dietary assessment tool, the Dietary Index for Gut Microbiota (DI-GM), which was developed based on intervention studies and encompasses 14 foods and nutrients closely linked to gut health. Unlike single biomarkers (e.g., *β*-glucuronidase activity or short-chain fatty acid levels), this comprehensive approach provides a more holistic reflection of dietary impacts on the gut microbiota.

However, this study has several limitations. First, dietary intake data were collected through 24-h dietary recall interviews or telephone interviews, which may be subject to recall bias. Second, respondents might have been influenced by social desirability bias, potentially underreporting the consumption of unhealthy foods such as high-sugar and high-fat products. Additionally, as this is a cross-sectional study, causal relationships between diet and gut microbiota cannot be established. Thus, the findings may be confounded by unmeasured factors, including lifestyle choices, genetic background, or psychological status. Future research should include prospective cohort studies to track dietary patterns and gut microbiota changes over time, as well as randomized controlled trials to assess the direct impact of dietary interventions on gut microbiota composition and related health outcomes.

## 6 Conclusion

This study systematically evaluated the association between the Dietary Index for Gut Microbiota (DI-GM) and female infertility using data from NHANES 2013–2018. The results revealed a negative association between DI-GM and the risk of female infertility, suggesting that optimizing dietary patterns to improve DI-GM scores could be an effective strategy for reducing the risk of female infertility.

## Data Availability

The original contributions presented in the study are included in the article/supplementary material, further inquiries can be directed to the corresponding authors.

## References

[1] Zegers-Hochschild F Adamson GD de Mouzon J Ishihara O Mansour R Nygren K. International Committee for Monitoring Assisted Reproductive Technology (ICMART) and the World Health Organization (WHO) revised glossary of ART terminology, 2009. Fertil Steril. (2009) 92:1520–4. doi: 10.1016/j.fertnstert.2009.09.009, PMID: 19828144

[2] Homan GF Davies M Norman R. The impact of lifestyle factors on reproductive performance in the general population and those undergoing infertility treatment: a review. Hum Reprod Update. (2007) 13:209–23. doi: 10.1093/humupd/dml056, PMID: 17208948

[3] Practice Committee of the American Society for Reproductive Medicine. Effectiveness and treatment for unexplained infertility. Fertil Steril. (2006) 86:S111–4. doi: 10.1016/j.fertnstert.2006.07.1475 17055802

[4] Luke B Brown MB Wantman E Lederman A Gibbons W Schattman GL. Cumulative birth rates with linked assisted reproductive technology cycles. N Engl J Med. (2012) 366:2483–91. doi: 10.1056/NEJMoa1110238, PMID: 22738098 PMC3623697

[5] Evers JL. Female subfertility. Lancet. (2002) 360:151–9. doi: 10.1016/s0140-6736(02)09417-5, PMID: 12126838

[6] Hertz-Picciotto I Dostál M Dejmek J Selevan SG Wegienka G Gomez-Caminero A. Air pollution and distributions of lymphocyte immunophenotypes in cord and maternal blood at delivery. Epidemiology. (2002) 13:172–83. doi: 10.1097/00001648-200203000-00012, PMID: 11880758

[7] Guo Y Qi Y Yang X Zhao L Wen S Liu Y. Association between polycystic ovary syndrome and gut microbiota. PLoS One. (2016) 11:e0153196. doi: 10.1371/journal.pone.0153196, PMID: 27093642 PMC4836746

[8] Wang Q Guan Z Guo X Chen B Li L Liu J. Stepwise Laparoendoscopic single-site Pectopexy for pelvic organ prolapse. J Minim Invasive Gynecol. (2021) 28:1142–3. doi: 10.1016/j.jmig.2020.10.008, PMID: 33096264

[9] DeGruttola AK Low D Mizoguchi A Mizoguchi E. Current understanding of Dysbiosis in disease in human and animal models. Inflamm Bowel Dis. (2016) 22:1137–50. doi: 10.1097/mib.0000000000000750, PMID: 27070911 PMC4838534

[10] O'Connor C Varshosaz P Moise AR. Mechanisms of feedback regulation of vitamin a metabolism. Nutrients. (2022) 14:1312. doi: 10.3390/nu14061312, PMID: 35334970 PMC8950952

[11] Zhang J Xu JH Qu QQ Zhong GQ. Risk of cardiovascular and cerebrovascular events in polycystic ovarian syndrome women: a Meta-analysis of cohort studies. Front Cardiovasc Med. (2020) 7:552421. doi: 10.3389/fcvm.2020.552421, PMID: 33282917 PMC7690560

[12] Pundir J Charles D Sabatini L Hiam D Jitpiriyaroj S Teede H. Overview of systematic reviews of non-pharmacological interventions in women with polycystic ovary syndrome. Hum Reprod Update. (2019) 25:243–56. doi: 10.1093/humupd/dmy045, PMID: 30608609

[13] Kase BE Liese AD Zhang J Murphy EA Zhao L Steck SE. The development and evaluation of a literature-based dietary index for gut microbiota. Nutrients. (2024) 16:1045. doi: 10.3390/nu16071045, PMID: 38613077 PMC11013161

[14] Depommier C Everard A Druart C Plovier H van Hul M Vieira-Silva S. Supplementation with *Akkermansia muciniphila* in overweight and obese human volunteers: a proof-of-concept exploratory study. Nat Med. (2019) 25:1096–103. doi: 10.1038/s41591-019-0495-2, PMID: 31263284 PMC6699990

[15] Rodríguez-Morató J Matthan NR Liu J de la Torre R Chen CO. Cranberries attenuate animal-based diet-induced changes in microbiota composition and functionality: a randomized crossover controlled feeding trial. J Nutr Biochem. (2018) 62:76–86. doi: 10.1016/j.jnutbio.2018.08.019, PMID: 30269035

[16] Blumberg JB Basu A Krueger CG Lila MA Neto CC Novotny JA. Impact of cranberries on gut microbiota and Cardiometabolic health: proceedings of the cranberry Health Research conference 2015. Adv Nutr. (2016) 7:759s–70s. doi: 10.3945/an.116.012583 27422512 PMC4942875

[17] Maukonen M Koponen KK Havulinna AS Kaartinen NE Niiranen T Méric G. Associations of plant-based foods, red and processed meat, and dairy with gut microbiome in Finnish adults. Eur J Nutr. (2024) 63:2247–60. doi: 10.1007/s00394-024-03406-x, PMID: 38753173 PMC11377619

[18] Zhang X Yang Q Huang J Lin H Luo N Tang H. Association of the newly proposed dietary index for gut microbiota and depression: the mediation effect of phenotypic age and body mass index. Eur Arch Psychiatry Clin Neurosci. (2024) 8:1–12. doi: 10.1007/s00406-024-01912-x, PMID: 39375215

[19] An S Qin J Gong X Li S Ding H Zhao X. The mediating role of body mass index in the association between dietary index for gut microbiota and biological age: a study based on NHANES 2007-2018. Nutrients. (2024) 16:164. doi: 10.3390/nu16234164, PMID: 39683559 PMC11643866

[20] Ye X Song X Zhou S Chen G Wang L. Association between combined healthy lifestyles and infertility: a cross-sectional study in US reproductive-aged women. BMC Public Health. (2025) 25:153. doi: 10.1186/s12889-025-21395-2, PMID: 39815248 PMC11734407

[21] Tong L Zhang X Chen J He H Zhang W Wan Z. The associations between dietary advanced glycation-end products intake and self-reported infertility in U.S. women: data from the NHANES 2013-2018. Sci Rep. (2025) 15:1158. doi: 10.1038/s41598-025-85361-z, PMID: 39774374 PMC11706955

[22] Moieni M Seeman TE Robles TF Lieberman MD Okimoto S Lengacher C. Generativity and social well-being in older women: expectations regarding aging matter. J Gerontol B Psychol Sci Soc Sci. (2021) 76:289–94. doi: 10.1093/geronb/gbaa022, PMID: 32064530 PMC7813180

[23] Lee R Mason A. Is low fertility really a problem? Population aging, dependency, and consumption. Science. (2014) 346:229–34. doi: 10.1126/science.1250542, PMID: 25301626 PMC4545628

[24] Akbaribazm M Goodarzi N Rahimi M. Female infertility and herbal medicine: An overview of the new findings. Food Sci Nutr. (2021) 9:5869–82. doi: 10.1002/fsn3.2523, PMID: 34646552 PMC8498057

[25] Wang S Gaskins AJ Farland LV Zhang D Birmann BM Rich-Edwards JW. A prospective cohort study of infertility and cancer incidence. Fertil Steril. (2023) 120:134–42. doi: 10.1016/j.fertnstert.2023.02.028, PMID: 36849034 PMC10293067

[26] Dybciak P Humeniuk E Raczkiewicz D Krakowiak J Wdowiak A Bojar I. Anxiety and depression in women with polycystic ovary syndrome. Medicina. (2022) 58:942. doi: 10.3390/medicina58070942, PMID: 35888661 PMC9319705

[27] Fitzpatrick JA Melton SL Yao CK Gibson PR Halmos EP. Dietary management of adults with IBD - the emerging role of dietary therapy. Nat Rev Gastroenterol Hepatol. (2022) 19:652–69. doi: 10.1038/s41575-022-00619-5, PMID: 35577903

[28] Herrera JL Ordoñez-Gutierrez L Fabrias G Casas J Morales A Hernandez G. Ovarian function modulates the effects of long-chain polyunsaturated fatty acids on the mouse cerebral cortex. Front Cell Neurosci. (2018) 12:103. doi: 10.3389/fncel.2018.00103, PMID: 29740285 PMC5928148

[29] Zhao G Tan Y Cardenas H Vayngart D Wang Y Huang H. Ovarian cancer cell fate regulation by the dynamics between saturated and unsaturated fatty acids. Proc Natl Acad Sci USA. (2022) 119:e2203480119. doi: 10.1073/pnas.2203480119, PMID: 36197994 PMC9564215

[30] Chen Q Wang B Wang S Qian X Li X Zhao J. Modulation of the gut microbiota structure with probiotics and Isoflavone alleviates metabolic disorder in Ovariectomized mice. Nutrients. (2021) 13:1793. doi: 10.3390/nu13061793, PMID: 34070274 PMC8225012

[31] Ohlsson C Lawenius L Andersson A Gustafsson K Wu J Lagerquist M. Mild stimulatory effect of a probiotic mix on bone mass when treatment is initiated 1.5 weeks after ovariectomy in mice. Am J Physiol Endocrinol Metab. (2021) 320:e591–7. doi: 10.1152/ajpendo.00412.2020, PMID: 33522399

[32] Salles BIM Cioffi D Ferreira SRG. Probiotics supplementation and insulin resistance: a systematic review. Diabetol Metab Syndr. (2020) 12:98. doi: 10.1186/s13098-020-00603-6, PMID: 33292434 PMC7656736

[33] Guo C Zhang C. Role of the gut microbiota in the pathogenesis of endometriosis: a review. Front Microbiol. (2024) 15:1363455. doi: 10.3389/fmicb.2024.1363455, PMID: 38505548 PMC10948423

[34] Pai AH Wang YW Lu PC Wu HM Xu JL Huang HY. Gut microbiome-Estrobolome profile in reproductive-age women with endometriosis. Int J Mol Sci. (2023) 24:16301. doi: 10.3390/ijms242216301, PMID: 38003489 PMC10671785

[35] Chen MJ Chou CH Hsiao TH Wu TY Li CY Chen YL. *Clostridium innocuum*, an opportunistic gut pathogen, inactivates host gut progesterone and arrests ovarian follicular development. Gut Microbes. (2024) 16:2424911. doi: 10.1080/19490976.2024.2424911, PMID: 39508647 PMC11545266

[36] Liu M Peng R Tian C Shi J Ma J Shi R. Effects of the gut microbiota and its metabolite short-chain fatty acids on endometriosis. Front Cell Infect Microbiol. (2024) 14:1373004. doi: 10.3389/fcimb.2024.1373004, PMID: 38938880 PMC11208329

[37] Salih Joelsson L Tydén T Wanggren K Georgakis MK Stern J Berglund A. Anxiety and depression symptoms among sub-fertile women, women pregnant after infertility treatment, and naturally pregnant women. Eur Psychiatry. (2017) 45:212–9. doi: 10.1016/j.eurpsy.2017.07.004, PMID: 28957789

[38] Juárez-Chairez MF Cid-Gallegos MS Meza-Márquez OG Jiménez-Martínez C. Biological functions of peptides from legumes in gastrointestinal health. A review legume peptides with gastrointestinal protection. J Food Biochem. (2022) 46:e14308. doi: 10.1111/jfbc.14308, PMID: 35770807

[39] Clark JL Taylor CG Zahradka P. Rebelling against the (insulin) resistance: a review of the proposed insulin-sensitizing actions of soybeans, chickpeas, and their bioactive compounds. Nutrients. (2018) 10:434. doi: 10.3390/nu10040434, PMID: 29601521 PMC5946219

[40] Casula E Pisano MB Serreli G Zodio S Melis MP Corona G. Probiotic lactobacilli attenuate oxysterols-induced alteration of intestinal epithelial cell monolayer permeability: focus on tight junction modulation. Food Chem Toxicol. (2023) 172:113558. doi: 10.1016/j.fct.2022.113558, PMID: 36528245

[41] Kaur I Suri V Sachdeva N Rana SV Medhi B Sahni N. Efficacy of multi-strain probiotic along with dietary and lifestyle modifications on polycystic ovary syndrome: a randomised, double-blind placebo-controlled study. Eur J Nutr. (2022) 61:4145–54. doi: 10.1007/s00394-022-02959-z, PMID: 35857132

[42] Liu M Kang Z Cao X Jiao H Wang X Zhao J. Prevotella and succinate treatments altered gut microbiota, increased laying performance, and suppressed hepatic lipid accumulation in laying hens. J Anim Sci Biotechnol. (2024) 15:26. doi: 10.1186/s40104-023-00975-5, PMID: 38369510 PMC10874536

[43] Deng W Wang H. Efficient cell chatting between embryo and uterus ensures embryo implantation†. Biol Reprod. (2022) 107:339–48. doi: 10.1093/biolre/ioac135, PMID: 35774025 PMC9310511

[44] Domar AD Seibel MM Benson H. The mind/body program for infertility: a new behavioral treatment approach for women with infertility. Fertil Steril. (1990) 53:246–9. doi: 10.1016/s0015-0282(16)53275-0, PMID: 2078200

[45] Wang T Sha L Li Y Zhu L Wang Z Li W. Combined Probiotic and Dietary Fiber Supplementation Improves Insulin Sensitivity and Restores Ovulation in Women With Polycystic Ovary Syndrome: A Randomized Controlled Trial. Front. Endocrinol. (2022) 11:284. doi: 10.3389/fendo.2020.00284, PMID: 36197994 PMC9564215

